# Comparative Studies of Vertebrate Beta Integrin Genes and Proteins: Ancient Genes in Vertebrate Evolution

**DOI:** 10.3390/biom1010003

**Published:** 2011-08-23

**Authors:** Roger S. Holmes, Ujjwal K. Rout

**Affiliations:** 1 School of Biomolecular and Physical Sciences, Griffith University, Nathan, 4111QLD, Australia; 2 Department of Surgery, University of Mississippi Medical Center, Jackson, MS 38677, USA; E-Mail: urout@umc.edu

**Keywords:** vertebrates, amino acid sequence, β-integrin, evolution, gene duplication

## Abstract

Intregins are heterodimeric α- and β-subunit containing membrane receptor proteins which serve various cell adhesion roles in tissue repair, hemostasis, immune response, embryogenesis and metastasis. At least 18 α- (ITA or ITGA) and 8 β-integrin subunits (ITB or ITGB) are encoded on mammalian genomes. Comparative ITB amino acid sequences and protein structures and *ITB* gene locations were examined using data from several vertebrate genome projects. Vertebrate *ITB* genes usually contained 13–16 coding exons and encoded protein subunits with ∼800 amino acids, whereas vertebrate *ITB4* genes contained 36-39 coding exons and encoded larger proteins with ∼1800 amino acids. The ITB sequences exhibited several conserved domains including signal peptide, extracellular β-integrin, β-tail domain and integrin β-cytoplasmic domains. Sequence alignments of the integrin β-cytoplasmic domains revealed highly conserved regions possibly for performing essential functions and its maintenance during vertebrate evolution. With the exception of the human ITB8 sequence, the other ITB sequences shared a predicted 19 residue α-helix for this region. Potential sites for regulating human *ITB* gene expression were identified which included CpG islands, transcription factor binding sites and microRNA binding sites within the 3′-UTR of human *ITB* genes. Phylogenetic analyses examined the relationships of vertebrate beta-integrin genes which were consistent with four major groups: 1: *ITB1, ITB2, ITB7*; 2: *ITB3, ITB5, ITB6*; 3: *ITB4*; and 4: *ITB8* and a common evolutionary origin from an ancestral gene, prior to the appearance of fish during vertebrate evolution. The phylogenetic analyses revealed that *ITB4* is the most likely primordial form of the vertebrate β integrin subunit encoding genes, that is the only β subunit expressed as a constituent of the sole integrin receptor ‘α6β4’ in the hemidesmosomes of unicellular organisms.

## 1. Introduction

Cell surface integrin receptors regulate cell-cell and cell-extra cellular matrix (ECM) interactions and are involved in mediating all known basic cellular processes (proliferation, migration, differentiation and death) in the body. Precise regulations of these cellular processes by a wide range of integrin receptors are witnessed in cells during development and later in life [1,2,3,4,5,6,7]. Disturbance of integrin function/s lead to suboptimal organogenesis in rodent animal models [5,6,7,8] and disease states in human populations [9,10].

An integrin receptor is a heterodimer consisting of an α and a β subunit, each containing extracellular, transmembrane and cytosolic domains. The extracellular domains of receptor subunits bind with the ECM proteins (such as fibronectin, laminin and collagen) and the cytosolic domains of β subunits interact with kinases (focal adhesion kinase and Src kinase), adaptor molecules (such as talin and kindlin) and the cytoskeleton (actin and microtubules) [5,11,12]. These interactions facilitate the ‘outside-in’ and the ‘inside-out’ signaling across the cell membrane by the integrin heterodimers [12,13,14].

While the evolutionary path of integrins in development and maintenance of cellular process are intensive areas of investigation [15,16,17], the evolution of different integrin subunits encoded within the vertebrate genomes remains to be fully elucidated. This knowledge is necessary for understanding the integrin receptors and the evolution of cellular functions that are coordinated by these versatile receptors. Evolution of integrin genes dates back to the time of transition of unicellular life forms into multicellular organisms [18,19]. It is known that the integrin-mediated adhesion system existed in the single celled *Amastigomonas* (Phylum *Apusozoa*), possibly for the purpose of attachment with the basal lamina, a transition towards sedentary life and multicellularity [19]. In vertebrates, a phylum that includes ∼53,000 species, the genes coding integrins are identified as early as in fishes (*Actinopterygians*) that evolved about 450 millions of years ago (Mya) [20]. Here we report the gene structures and amino acid sequences for vertebrate β-integrin encoding genes (*ITB*) and proteins (ITB), respectively, as well as their phylogenetic and evolutionary relationships. Potential regulatory sites for several human *ITB* genes, predicted secondary structures of signal peptides and cytoplasmic domains and tissue specific expression for mammalian ITB genes are also discussed in terms of their homology and evolution.

## 2. Results and Discussion

### 2.1. Vertebrate ITB Gene Locations and Exonic Structures

Table 1 summarizes the locations and predicted structures for vertebrate *ITB* genes based upon BLAT interrogations of several vertebrate genomes using the reported sequences for human ITB1 [21,22,23,24], ITB2 [25,26,27]; ITB3 [28,29,30,31]; ITB4 [32,33,34]; ITB5 [35,36,37]; ITB6 [38,39,40]; ITB7 [41,42]; and ITB8 [43,44] and the University of California Santa Cruz (UCSC) Genome Browser [45]. The predicted vertebrate *ITB* genes predominantly contained 13–16 coding exons, with the exception of vertebrate *ITB4* genes which exhibited 36 (opossum *ITB8*) to 39 coding exons and encoded larger ITB protein subunits (∼1,800 amino acids) as compared with other ITB subunits which contained ∼800 amino acids in sequence (Table 1). *ITB* genes were separately located on vertebrate chromosomes for each of the genomes examined in comparison with other gene families which may be clustered on a single chromosome (e.g., the alcohol dehydrogenase (*ADH*) gene family) [46] or a small number of chromosomes such as the lactate dehydrogenase (*LDH*) gene family [47].

### 2.2. Vertebrate ITB Signal Peptides and Domain Structures

Application of the SignalP 3.0 server predicted a standard length of signal peptides for ITB1 (20 aa), ITB2 (22 aa), ITB3 (26 aa), ITB4 (27 aa), ITB5 (24 aa), ITB6 (21 aa) and ITB7 (19 aa) subunits except for a long signal sequence (42 aa) for the smallest size integrin isoform ITB8. Although, the server provided a distinct cleavage site for signal peptides in all human ITB forms, a recent study has shown that the signal peptide of the β2 integrin subunit in ruminants containing cleavage inhibition glutamine (Q) was not processed [48]. Not much is known about the signal peptide processing of integrins; however, human ITB1 and ITB2 integrin subunits contain cleavage inhibiting ‘Q’ at the predicted cleavage sites (data not shown). Domain annotation of signal sequences of ITB genes predicted a central helical domain with an anterior and a posterior coiled motifs in ITB1, ITB2, ITB3, ITB5, ITB6 and ITB7 subunits. The signal peptide for ITB8, however, consisted of two central helical motifs separated by a coiled motif and two additional coiled motifs at the N and C terminal ends of the signal peptide. The predicted signal peptide sequences from different ITB subunits showed little evidence of sequence similarity (data not shown) that is not uncommon for signal peptides [49]. The lack of identity amongst the primary structures of signal sequences of different ITB subunits and the similarity amongst the secondary structures (a central hydrophobic core with coiled motifs at the ends) implies that these secondary structures of signal sequences are indispensible conformations for the insertion of the *N*-terminal ends of beta-subunits into the cell membrane. The reason for the very long signal sequence and two hydrophobic motifs in the ITB8 structure is unclear although it is possible that an additional hydrophobic motif may enhance the processing and translocation of ITB8 into the lipid bilayer [50,51].

**Figure 1:**
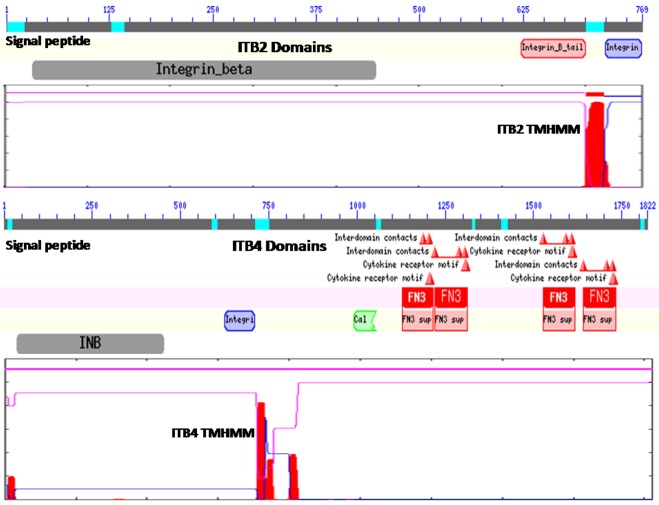
Predicted domains and transmembrane helix for human ITB2 and ITB4. Domains and key regions are identified for ITB2 and ITB4 amino acid sequences using NCBI web tools (http://www.ncbi.nlm.nih.gov/Structure/cdd/wrpsb.cgi) to identify functional domains and ExPasy web tools to identify predicted transmembrane domains (TMHMM) (http://www.cbs.dtu.dk/services/TMHMM-2.0/); INB or integrin_beta; integrin B tail (in pink); TMHMM transmembranes (in red); cytosolic domain (in blue); Calx-beta domain (in green); FN3 (fibronectin 3), cytokine receptor and interdomain contacts (red triangles); Note: lack of FN3 binding domains and the cytokine receptor motifs in β4 subunit that interacts only with laminin-332.

Figure 1 illustrates the predicted domain structures for ITB2 and ITB4, with the former representing the domain structures for ITB1, ITB3, ITB5, ITB6, ITB7 and ITB8 [52] including the *N*-signal peptide previously described (residues 1–22 for ITB2); an extracellular integrin beta region (pfam00362) (residues 32–447) including a potential cell attachment site (residues 397–399) and a region of cysteine-rich tandem repeats (residues 414–617); an integrin beta tail domain (pfam07965) (residues 622–700); a transmembrane helical region (residues 701–723) (see Figure 1 for ITB2 TMHMM region), which anchors ITB2 to the cell membrane; and an ITB2 cytoplasmic region (residues 724–768).

**Table 1:** Vertebrate beta integrin and nematode beta integrin-like genes and proteins. RefSeq: the reference amino acid sequence; ^1,3^ predicted Ensembl amino acid sequence; ^2^ not available; ^4^ Contig refers to a DNA scaffold for sequencing analyses; GenBank IDs are derived NCBI http://www.ncbi.nlm.nih.gov/genbank/; Ensembl ID was derived from Ensembl genome database http://www.ensembl.org/; UNIPROT refers to UniprotKB/Swiss-Prot IDs for individual acid lipases (see http://kr.expasy.org/); bps refers to base pairs of nucleotide sequences; pI refers to theoretical isoelectric points; the number of coding exons are listed.

**Animal**	**Species**	**Integrin Beta Gene (subunit)**	**Other Gene Name**	**Chromosome Coordinates**	**Gene Size (kbps)**	**Coding Exons (Strand)**	**Subunit MW**	**Amino Acids**	**NCBI^1,2^ ID**	**UNIPROT^2^ ID**
Human	*Homo sapiens*	*ITB1 (*β*1)*	*ITGB1*	10:33,190,501-33,224,486	35.2	16 (−)	88,415	798	NM_002211	P05556
Mouse	*Mus musculus*	*Itb1 (*β*1)*	*Itgb1*	8:131,209,552-131,257,438	47.9	15 (+)	88,231	798	NM_010578	P09055
Horse	*Equus caballus*	*ITB1 (*β*1)*	*ITGB1*	29:5,050,336-5,076,608	26.3	15 (+)	88,202	798	XP_001492715^2^	na
Opossum	*Monodelphis domestica*	*ITB1 (*β*1)*	*ITGB1*	8:240,074,436-240,104,533	30.1	15 (+)	88,329	799	XP_001366567^2^	na
Chicken	*Gallus gallus*	*ITB1 (*β*1)*	*ITGB1*	2:13,977,440-14,000,322	22.9	15 (+)	88,554	803	NP_001034343	P07228
Frog	*Xenopus tropicalis*	*ITB1 (*β*1)*	*ITGB1*	503^5^:452,582-465,188	12.6	15 (+)	88,083	798	NP_989160	Q6P4X1
Zebrafish	*Danio rerio*	*ITB1A (*β*1A)*	*ITGB1A*	24:1,010,009-1,028,526	18.5	15 (−)	88,592	798	NP_001030143	Q3YAA1
Zebrafish	*Danio rerio*	*ITB1B (*β*1B)*	*ITGB1B*	2:42,692,236-42,708,345	16.1	15 (+)	86,570	787	NP_001030151	Q3YA99
Human	*Homo sapiens*	*ITB2 (*β*2)*	*ITGB2*	21:46,306,286-46,330,697	40.0	15 (−)	84,782	769	NM_001127491	P05107
Mouse	*Mus musculus*	*Itb2 (*β*2)*	*Itgb2*	10:76,993,093-77,028,419	35.3	15 (+)	85,026	771	NM_008404	P11835
Mouse	*Mus musculus*	*Itb2l (*β*2l)*	*Itgb2l*	16:96,643,905-96,665,221	21.3	15 (−)	81,547	738	NM_008405	Q3UV74
Horse	*Equus caballus*	*ITB2A (*β*2A)*	*ITGB2A*	26:39,992,918-40,009,865	16.9	15 (−)	85,290	770	XP_001490052^2^	na
Horse	*Equus caballus*	*ITB2B (*β*2B)*	*ITGB2B*	26:40,054,164-40,069,857	15.7	14 (−)	79,824	726	chr26.199.1^3^	na
Opossum	*Monodelphis domestica*	*ITB2 (*β*2)*	*ITGB2*	2:539,079,162-539,090,540	11.4	13 (−)	83,015	761	chr2.11.580.a^3^	na
Chicken	*Gallus gallus*	*ITB2 (*β*2)*	*ITGB2*	7:7,143,899-7,150,094	6.2	15 (−)	85,409	772	NP_990582	na
Frog	*Xenopus tropicalis*	*ITB2 (*β*2)*	*ITGB2*	2185:1,356,257-1,405,734	49.5	16 (−)	86,980	782	XP_002936570^2^	na
Zebrafish	*Danio rerio*	*ITB2 (*β*2)*	*ITGB2*	9:47,603,423-47,619,406	16.0	15 (+)	84519	768	XP_686012^2^	na
Human	*Homo sapiens*	*ITB3 (*β*3)*	*ITGB3*	17:45,331,228-45,387,567	37.4	9 (+)	87,058	788	NM_000212	P05106
Mouse	*Mus musculus*	*Itb3 (*β*3)*	*Itgb3*	11:104,469,370-104,528,689	59.3	15 (+)	86,694	787	NM_016780	O54890
Horse	*Equus caballus*	*ITB3 (*β*3)*	*ITGB3*	11:17,206,504-17,237,153	30.7	15 (−)	86,360	784	NM_001081802	na
Opossum	*Monodelphis domestica*	*ITB3 (*β*3)*	*ITGB3*	2:208,102,619-208,154,477	51.9	14 (+)	84,497	764	chr2.5.154.a^3^	na
Chicken	*Gallus gallus*	*ITB3 (*β*3)*	*ITGB3*	27:2,207,723-2,223,943	16.2	15 (+)	86,088	781	NP_989646	na
Frog	*Xenopus tropicalis*	*ITB3 (*β*3)*	*ITGB3*	973^5^:169,881-200,256	30.4	15 (−)	88,235	792	XP_002942401^3^	na
Zebrafish	*Danio rerio*	*ITB3A (*β3A*)*	*ITGB3A*	3:16,152,931-16,181,252	28.3	15 (+)	85,693	785	NP_001032312	Q3LTM4
Zebrafish	*Danio rerio*	*ITB3B (*β*3B)*	*ITGB3B*	12:21,495,417-21,520,656	25.2	15 (−)	87,700	790	NP_001076417	B3DIP9
Human	*Homo sapiens*	*ITB4 (*β*4)*	*ITGB4*	17:73,720,784-73,753,633	36.4	39 (+)	202,167	1,822	NM_000213	P16144
Mouse	*Mus musculus*	*Itb4 (*β4*)*	*Itgb4*	11:115,836,039-115,869,725	33.7	39 (+)	201,650	1,818	NM_133663	A2A863
Horse	*Equus caballus*	*ITB4 (*β*4)*	*ITGB4*	11:6,286,192-6,312,590	26.4	39 (−)	194,864	1,752	XP_001915915^2^	na
Opossum	*Monodelphis domestica*	*ITB4 (*β*4)*	*ITGB4*	2:213,276,209-213,319,767	43.6	36 (−)	198,824	1,778	XP_001377606^2^	na
Chicken	*Gallus gallus*	*ITB4 (*β*4)*	*ITGB4*	18:4,713,504-4,732,758	19.3	39 (−)	203,330	1,818	E1C9G7^4^	E1C9G7
Frog	*Xenopus tropicalis*	*ITB4 (*β*4)*	*ITGB4*	545^5^:495,511-545,134	49.6	38 (+)	204,208	1,835	XP_002940020^2^	na
Zebrafish	*Danio rerio*	*ITB4 (*β*4)*	*ITGB4*	8:13,034,599-13,080,896	46.3	39 (+)	210,736	1,893	NP_001019557	Q4U0S1
Human	*Homo sapiens*	*ITB5 (*β*5)*	*ITGB5*	3:124,482,473-124,605,847	139.5	15 (−)	88,054	799	BC006541^6^	P18084
Mouse	*Mus musculus*	*Itb5 (*β*5)*	*Itgb5*	3:126,102,954-125,963,482	139.5	15 (+)	87,909	798	NM_010580	O70309
Horse	*Equus caballus*	*ITB5 (*β*5)*	*ITGB5*	19:34,769,099-34,876,312	107.2	15 (+)	87,985	803	XP_001500077^2^	na
Opossum	*Monodelphis domestica*	*ITB5 (*β*5)*	*ITGB5*	4:90,575,064-90,751,974	17.7	14 (−)	86,072	779	XP_001372711^2^	na
Chicken	*Gallus gallus*	*ITB5 (*β*5)*	*ITGB5*	7:29,463,064-29,500,494	37.4	15 (−)	88,475	812	NP_989814	na
Frog	*Xenopus tropicalis*	*ITB5 (*β*5)*	*ITGB5*	6	6	6	87,561	807	NP_001135704	B5DEV6
Zebrafish	*Danio rerio*	*ITB5 (*β*5)*	*ITGB5*	9:22,031,538-22,101,156	69.6	16 (+)	88,956	802	NP_001076305	A5D6V1
Human	*Homo sapiens*	*ITB6 (*β*6)*	*ITGB6*	2:160,958,250-161,056,574	172.2	15 (−)	85,936	788	NM_000888	P18564
Mouse	*Mus musculus*	*Itb6 (*β*6)*	*Itgb6*	2:160,836,644-160,664,416	172.2	15 (+)	86,042	787	NM_021359	Q9Z0T9
Horse	*Equus caballus*	*ITB6 (*β*6)*	*ITGB6*	18:40,790,052-40,860,613	70.6	15 (−)	85,817	788	XP_001492914^2^	na
Opossum	*Monodelphis domestica*	*ITB6 (*β*6)*	*ITGB6*	4:165,626,656-165,720,419	93.8	15 (−)	86,274	787	ENSMODT6582^3^	na
Chicken	*Gallus gallus*	*ITB6 (*β*6)*	*ITGB6*	7:23,382,214-23,409,883	27.7	15 (+)	86,530	789	XP_422037^2^	E1C6K8
Frog	*Xenopus tropicalis*	*ITB6 (*β*6)*	*ITGB6*	51^5^:1,605,719-1,638,193	32.5	13 (+)	76,152	696	NP_001090775	A4IGI8
Human	*Homo sapiens*	*ITB7 (*β*7)*	*ITGB7*	12:53,585,343-53,594,227	16.3	15 (−)	86,903	798	NM_000889	P26010
Mouse	*Mus musculus*	*Itb7 (*β7*)*	*Itgb7*	15:102,046,428-102,062,317	15.9	15 (−)	87,411	806	NM_013566	P26011
Horse	*Equus caballus*	*ITB7 (*β*7)*	*ITGB7*	6:70,193,060-70,201,400	8.3	14 (−)	86,666	797	XP_001494917^2^	na
Frog	*Xenopus tropicalis*	*ITB7 (*β*7)*	*ITGB7*	226:1,387,775-1,412,224	24.5	15 (+)	84,461	766	XP_002936686^2^	na
Zebrafish	*Danio rerio*	*ITB7 (*β*7)*	*ITGB7*	6:43,251,691-43,271,173	19.5	15 (−)	74,006	661	XP_001337949^2^	na
Human	*Homo sapiens*	*ITB8 (*β*8)*	*ITGB8*	7:20,371,430-20,449,617	85.1	14 (+)	85,632	769	NM_002214	P26012
Mouse	*Mus musculus*	*Itb8 (*β8*)*	*Itgb8*	12:120,477,276-120,396,490	80.8	14 (−)	84,519	767	NM_177290	Q0VDB0
Horse	*Equus caballus*	*ITB8 (*β*8)*	*ITGB8*	4:52,216,892-52,290,256	73.4	14 (+)	85,361	767	XP_001497271^2^	na
Opossum	*Monodelphis domestica*	*ITB8 (*β8*)*	*ITGB8*	8:302,826,175-302,899,169	73.0	14 (−)	83,721	757	XP_001371834^2^	na
Chicken	*Gallus gallus*	*ITB8 (*β8*)*	*ITGB8*	2:30,002,182-30,052,908	49.3	15 (+)	86,990	786	XP_418706^2^	na
Frog	*Xenopus tropicalis*	*ITB8 (*β8*)*	*ITGB8*	52^5^:945,865-97,887	33.0	14 (−)	81,104	728	XP_002933344^2^	na
Zebrafish	*Danio rerio*	*ITB8 (*β8*)*	*ITGB8*	19:2,377,677-2,441,753	64.1	14 (−)	74,877	669	XP_001919626^2^	A3RI57
Nematode	*Caenorhabditis elegans*	*PAT3*	*pat3*	III:3,909,309-3,914,106	4.8	8 (−)	90,138	809	NP_497787	Q27874

In contrast, ITB4 is a much larger protein compared with the other beta-integrins (1822 residues compared with 769–798 residues) and contains several beta-integrin-like domains in the *N*-terminal half of the protein [12,53,54] including the *N*-signal peptide (residues 1–27); an *N*-terminal extracellular beta-integrin domain (residues 37–453); a cysteine-rich tandem-repeat region (residues 456–619); an integrin beta-tail region (residues 626–711); and an ITB4 transmembrane-helix anchor region (residues 711–733) (Figure 1). The cytoplasmic region of ITB4 contains four fibronectin type III (FN3) domains (residues 1128–1215; 1220–1313; 1528–1628; and 1641–1734) and a Calx-beta motif (residues 991–1054) which are responsible for most of the intracellular interactions of the integrin [53,54,55,56]. Other key cytoplasmic ITB4 domains include several interdomain contact sites (residues 1641, 1708 and 1723) and cytokine-receptor motifs (1608–1609 and 1611–1612).

### 2.3. Alignments of Vertebrate ITB Cytosolic Isoform Sequences

**Figure 2:**
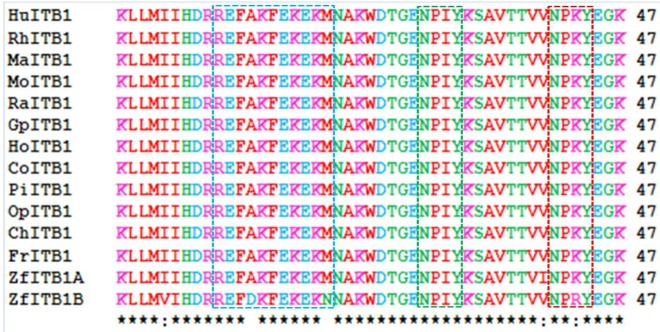
Amino acid alignments for vertebrate ITB1 cytosolic domain sequences. ITB1 sequences examined included Hu-human; Rh-rhesus; Ma-marmoset; Mo-mouse; Ra-rat; Gp-guinea pig; Ho-horse; Co-cow; Pi-pig; Op-opossum; Ch-chicken; Fr-*Xenopus tropicalis*; Zf-zebrafish; see Table 1 for details; note that 2 ITB1-like genes were observed in zebrafish (designated as ITB1A and ITB1B); * shows identical residues for ITB subunits; : similar alternate residues;. dissimilar alternate residues; α-helix for vertebrate ITB sequences is in shaded yellow; β-sheet is in shaded grey; colors for amino acids are shown as: **basic** (R and K); **acidic** (D and E); **neutral hydrophilic** (G, Y, Q, S, T, N, Y, C, H); and **hydrophobic** (M, A, F, I, L, W, P, V); the Cyto-1, Cyto-2 (NPXY) and Cyto-3 (NXXY) domains are shown in dotted lines (see text for details).

The cytoplasmic domains of β subunits interact with several intracellular proteins. Many of these interactions are known to cause conformational change in the extracellular domain changing the affinity of the receptor with the ECM. The interactions of extracellular domains with the ECM may also cause change in the conformation of the cytosolic domain allowing its interaction with the non-receptor tyrosine kinases and the actin cytoskeleton. Therefore, the cytoplasmic domain of integrin β subunits plays crucial roles in both ‘outside-in’ and ‘inside-out’signaling [12,13,14]. Figure 2 examines alignments of vertebrate ITB1 cytosolic domain sequences which are color coded for amino acid residue properties. With the exception of a second duplicated ITB1.1 (designated as *ITB1B*) gene product observed in zebrafish (*Danio rerio*), identical sequences were observed for the ITB1 cytosolic domain for all vertebrates examined, which indicates that this is a highly conserved region of ITB1 which undertakes essential functions and is subject to selection and maintenance of this sequence. Comparisons of the cytosolic domain sequences for the other ITB proteins (alignments not shown) revealed lower levels of amino acid sequence identities as compared with the highly conserved ITB1 cytosolic domain sequence: ITB2 (37% identities); ITB3 (77% excluding the gene duplicate product ITB3B from zebrafish); ITB5 (50%); ITB6 (74%); ITB7 (35%); and ITB8 (45%).

**Figure 3:**
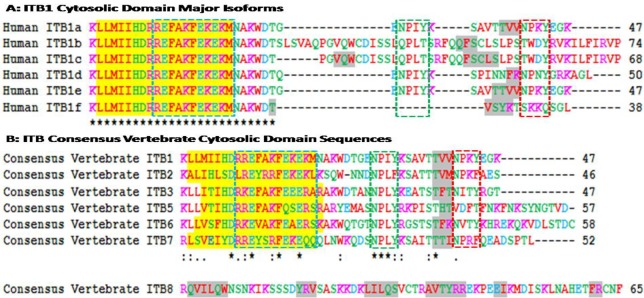
Amino acid sequence alignments for vertebrate ITB cytosolic domain sequences. **(A)** Comparison and alignments of human ITB1 major isoforms for cytosolic domain sequences; (**B)** Consensus sequences of vertebrate ITB cytosolic domains; see Table 1 for sources of beta integrin cytosolic domain sequences: * shows identical residues for ITB subunits; : similar alternate residues;. dissimilar alternate residues; α-helix for vertebrate ITB sequences is in shaded yellow; β-sheet is in shaded grey; colors for amino acids are shown as: **basic** (R and K); **acidic** (D and E); **neutral hydrophilic** (G, Y, Q, S, T, N, Y, C, H); and **hydrophobic** (M, A, F, I, L, W, P, V); the Cyto-1, Cyto-2 (NPXY) and Cyto-3 (NXXY) domains are shown in dotted lines (see text for details).

Figure 3A shows amino acid sequence alignments for the six major human ITB1 isoforms designated as ITB1a-ITB1f [57]. Residues 1–26 were identical for each of the isoforms which contained the 19 residue α-helix region, whereas the *C*-terminal differed in length and sequence and exhibited 1–2 predicted β-sheet regions. Recent studies [58] have shown that ITB1a is expressed in fetal muscles but is substituted by ITB1d during postnatal development. The *C*-terminal region is exposed at the cytoplasmic face of the plasma membrane where it is bound to the actin filaments. ITB1d is expressed only in striated muscle tissues and binds to both cytoskeletal and extracellular matrix proteins with an affinity higher than ITB1a which provides a stronger link between the cytoskeleton and extracellular matrix to support mechanical tension during muscle contraction. ITA1a and ITA1b have been shown to be similar as far as the alpha/beta association and fibronectin binding are concerned but differ, however, in their subcellular localization. ITB1a has been localized in focal adhesions whereas ITBb does not and exhibits distinct properties [22]. Human ITB1 isoforms are differentially expressed in tissues and exhibit distinct binding properties. HumanITB1a is widely expressed and usually coexpressed with other isoforms with a more restricted distribution. ITB1b is expressed in skin, liver, skeletal muscle, cardiac muscle, placenta, umbilical vein endothelial cells, neuroblastoma cells, lymphoma cells, hepatoma cells and astrocytoma cells. ITB1c is expressed in muscle, kidney, liver, placenta, cervical epithelium, umbilical vein endothelial cells, fibroblast cells, embryonic kidney cells, platelets and several blood cell lines, whereas ITB1d is expressed specifically in striated muscle (skeletal and cardiac muscle).

Amino acid sequence alignments for vertebrate consensus sequences ITB cytosolic domains are shown in Figure 3B. Human ITB sequences were based on previous reports for ITB1 [21,22,23,24]; ITB2 [25,26,27]; ITB3 [28,29,30,31]; ITB5 [35,36,37]; ITB6 [38,40]; ITB7 [39,41,42]; and ITB8 [43,44]. With the exception of the human ITB8 sequence, the other ITB sequences were of similar length (46-58 amino acids) and shared a predicted 19 residue α-helix region (residues 2–20 for human ITB1) whereas the human ITB8 sequence contained 65 amino acids with 6 predicted β-sheets.

The cytoplasmic tail of the ITB4 subunit is exceptionally long (1072 residues) compared to other ITB subunits [33] that are much shorter (Figure 3). Point mutation analysis of the cytoplasmic sequences of these β integrin subunits reveal three clusters of amino acids in the β cytoplasmic tail that regulate the interaction of integrins with the cytoskeleton, localization of receptors at the adhesion complex and inside-out signaling [12,13,59,60,61,62]. These three clusters of amino acids (Signalins) are commonly known as cyto 1, cyto-2 and cyto-3: cyto-1 is present in the vicinity of transmembrane domain, whereas cyto-2 (NPXY motif) and cyto-3 (NXXY motif) are located in the proximal and distal regions respectively of a tail (Figure 2) [63]. Alignment results of ITB1 subunits of different species (Figure 2) and spliced versions of ITB1 subunits (Figure 3A) show that the cyto-1 residues remain highly conserved indicating their conservation during vertebrate evolution for their specificity in function. Recent studies have shown that the interaction between the conserved arginine residue in the α-tail and aspartate residue in the β-tail, and by the hydrophobic residues immediately *N*-terminal to the arginine and aspartate residues play important role in ‘inside-out’ signalling by forming a ‘clasp’ between the α and β subunits [64,65].

The cyto-3 sequence in contrary varied amongst the spliced versions of ITB1 and the different ITB subunits (Figure 3). Therefore, the variability in the functions of different spliced versions of ITB1 (Figure 3A) and amongst different ITB isoforms (Figure 3B) may be derived from the differences in the cyto 2 and cyto 3 sequences. Moreover, each β subunit conceals distinct differences in its affinity towards intracellular proteins that is shown to be dictated by the ‘X’ and the neighboring amino acids of these motifs [66]. For instance, the binding of ICAP-1α, a 200amino acid protein, with the cyto-3 is influenced by the proximal Val787 and Val 790 [66,67,68]. The NPXY and NXXY motifs, with the propensity to form β turns, act as canonical recognition sequences of intracellular proteins with phosphotyrosine-binding domains (PTB) [66]. These include the interaction of β1A tail with the PTB domain of talin, EPS8 and Dab1; β2 tail with Dok-1 and talin; β3 tail with Numb, Dab1, EPS8, Tensin, Dok-1 and talin; β5 tail with Numb, Dab1, Dab2, EPS8, Tensin, Dok-1 and talin and the β7 tail with tensin, Dok-1 and Talin [67].

Recent studies have reported that cytosolic proteins kindlin-1, 2 and 3 are essential for integrin activation [68,69]. Immuno-precipitation assays with β integrin tails show that isoforms of kindlins bind with membrane proximal NPXY and membrane distal NXXY motifs as well as neighboring residues (NPXY linker region) of the β integrin subunit [69]. Several other cytosolic proteins (including filamin, melusin and myosin) also bind both conserved and non-conserved domains of β cytoplasmic tails [70]. Therefore, differences in the residues within and around these motifs in vertebrate β integrin subunits may change the affinities of these cytosolic proteins with the β integrin tail. Phosphorylation of the tyrosine residue in the distal NXXY motif of the β3 subunit disrupts the recognition by kindlin-2 and co-activation of aIIb.b3 integrin by talin [71,72]. The phosphoryalated or unphosphorylated state of tyrosines may also determine the affinities of proteins with the cytosolic tail. The unphosphorylated state of Y747 in the of β3 integrin tail has a 3 fold preference for the talin over the PTB domain of Dok1, whereas with the phosphorylated state of Y747, this affinity is increased 400 fold for Dok1 and decreased 2 fold for talin [73]. A recent study shows that phosphorylation of tyrosine 759 inhibits binding of kindling-2 with the *C*-terminal β3 chain [71]. Thus the expression patterns of different β subunits (Figure 5) and interacting proteins in the cytosol as well the phosphorylation state of ‘Y’ may determine the functional output of the integrin receptors.

### 2.4. Human ITB genes: Introns, Isoforms and Predicted Regulatory Regions

**Figure 4:**
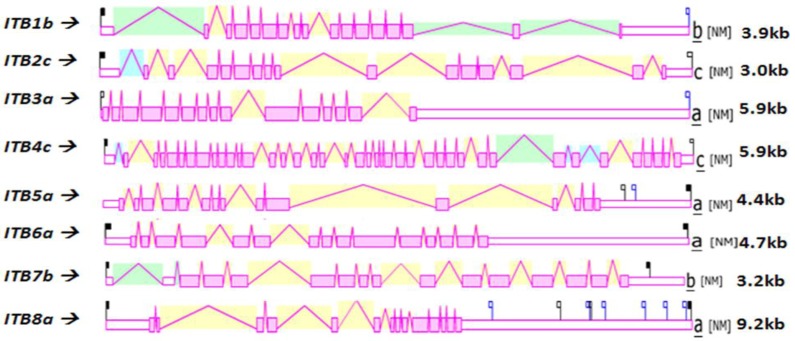
Structures and major splicing isoforms for human beta integrin genes. Derived from the AceView website http://www.ncbi.nlm.nih.gov/IEB/Research/Acembly/ [57]; mature isoform variants (a) are shown with capped 5′- and 3′- ends for the predicted mRNA sequences; NM refers to the NCBI reference sequence; exons are in pink; the directions for transcription are shown as 5′ → 3′; sizes of mRNA sequences are shown in kilobases (kb).

Figure 4 shows the predicted structures of mRNAs for human *ITB* transcripts for the major isoform in each case [57]. The transcripts were 3.0–9.2 kbs in length and exhibited distinct exonic structures in each case, including extended 3′-untranslated regions (UTR), especially for *ITB3a, ITB6a* and *ITB8a* transcripts. The number of *ITB* introns varied widely among the vertebrate genes examined: the *ITB4* gene contained the largest number of introns (39) followed by *ITB1* (16), *ITB2* and *ITB7* (15), *ITB3, ITB5* and *ITB6* (14) and *ITB8* (13).

The human *ITB* genome sequences contained several predicted transcription factor binding sites (TFBS), microRNA sites located in the 3′-untranslated region and CpG islands, which included CpG158, CpG92, CpG91, CpG152 and CpG133 located in the 5′-untranslated region of human *ITB1*, *ITB3*, *ITB4*, *ITB5* and *ITB8*, respectively (see Table 2). These CpG islands within the *ITB* gene promoters may play major contributing roles in maintaining high levels of gene expression (1.4–6.1 times the average for human genes) [57] which are similar to CpG islands within housekeeping gene promoters expressed in most tissues [74]. Large numbers of TFBS sites were observed for most of the human *ITB* genes examined, including 51, 56 and 105 such sites for *ITB4, ITB6* and *ITB8,* respectively. Of particular significance for the human *ITB1* and *ITB3* gene promoters is the transcription factor, HoxD3, that binds directly to these promoters and assists in regulating the expression of integrins α5β1 and αVβ3 during angiogenesis [75]; the PPARα (peroxisome proliferator-activated receptor-α) that regulates gene expression in vascular cells and inhibits TGF (transforming growth factor)- β-induced *ITB5* transcription [76] and Hox A10, that directs the regulation of the *ITB3* gene in human endometrial cells and regulates transcription of ITB3 during myeloid differentiation [77,78]. Moreover, the genes encoding the integrin subunits β7, β3, β6 and β8 map to 12q13.13, 17q21.32, 2q23-q31 and 7p15-p21 positions respectively which are close to *HOXC, HOXB, HOXD* and *HOXA* genes suggesting a common divergence of these genes during vertebrate evolution [79].

**Table 2:** Predicted transcription factor binding sites (TFBS), CpG islands and MiRNA (MiR) regions for human and mouse *ITB* Genes. The human and mouse genome browsers (http://genome.ucsc.edu) [45] were used to examine the predicted transcription factor binding sites (TFBS), CpG islands and Mi-RNA binding sites for human and mouse ITB genes.

**Animal**	**Species**	**Integrin Beta Gene**	**Other Gene Name**	**CpG Islands**	**TFBS**	**MiR-Sites 3′ region**	**Expression Level (x average)**	**Major Tissue Expression**
Human	*Homo sapiens*	*ITB1*	*ITGB1*	CpG:158	39	8	6.1	wide expression
		*ITB2*	*ITGB2*	CpG18,21,22	7	0	3.8	thymus, spleen, bone
		*ITB3*	*ITGB3*	CpG92	10	5	1.4	placenta, kidney, skin
		*ITB4*	*ITGB4*	CpG19,22,62,91	51	2	4.6	colon, ovary, pancreas
		*ITB5*	*ITGB5*	CpG152	47	0	4.5	lung, ovary, kidney
		*ITB6*	*ITGB6*	0	56	0	1.3	pancreas, lung, kidney
		*ITB7*	*ITGB7*	CpG48	20	0	0.8	leukocytes, spleen
		*ITB8*	*ITGB8*	CpG133	105	35	2.2	wide expression

Mouse	*Mus musculus*	*Itb1*	*Itgb1*	CpG120	na	3	2.0	wide expression
		*Itb2*	*Itgb2*	0	na	0	1.6	thymus, spleen, bone
		*Itb2l*	*Itgb2l*	0	na	0	0.3	bone marrow
		*Itb3*	*Itgb3*	CpG522	na	3	0.5	spleen, placenta
		*Itb4*	*Itgb4*	CpG38,59	na	1	1.8	mammary, brain
		*Itb5*	*Itgb5*	CpG87	na	-	3.7	mammary, lung
		*Itb6*	*Itgb6*	0	na	1	0.4	kidney, bladder, heart
		*Itb7*	*Itgb7*	CpG26	na	0	1.1	spleen, mammary
		*Itb8*	*Itgb8*	CpG316	na	2	1.2	mammary, kidney

Several microRNA (miRNA) binding sites within the 3′-untranslated region (3′-UTR) of human *ITB* mRNA were also identified (Table 2). These microRNA species are phylogenetically conserved and regulate mRNA and protein expression during embryonic development [80,81]. MiRNA 183, for example, inhibits tumor invasiveness and participates in the development and function of neurosensory organs by targeting the *ITB1* (mRNA) gene [82] whereas *ITB3* gene expression is apparently regulated by miRNA let-7a in malignant melanoma [83]. Over-expression of mir-124 attenuates endogenous *ITB1* expression in oral squamous cell carcinomas [84] while microRNA miR-93 promotes tumor growth and angiogenesis by decreasing ITB8 transcripts [85]. The number of microRNAs that target the 3′ UTR of human *ITB* transcripts (4 for *ITB1*, 44 for *ITB2*, 5 for *ITB3*, 1 for *ITB4*, 53 for *ITB5*, 11 for *ITB6*, 30 for *ITB7* and 2 for *ITB8*) varies widely among the human *ITB* genes examined. The absence of redundancy among this wide range of microRNA species regulating the levels of human integrin subunits suggests that the evolution of the *C*-terminal non-coding regions of these subunits followed a divergent path for the purpose of regulating the levels of expressions of each ITB subunits in different cells. The regulation of *ITB4* by a single microRNA further suggests that the expression of this subunit is not intensely regulated at the post-transcriptional stage in comparison with the other human *ITB* genes.

Brendle and coworkers [86] have also examined single nucleotide polymorphisms (SNPs) in predicted miRNA sites for several *ITA* and *ITB* genes and the potential association of these SNPs with breast cancer risk (BCR) and reported a potential BCR marker for one of the *ITB4* miRNA binding sites. A likely mechanism for mi-RNA translational regulation has been recently reported [87]. MicroRNAs have been shown to be transcribed as long primary-miRNAs (pri-miRNAs) in the nucleus and processed in the cytoplasm into 19-22 bp mature mi-RNAs which anneal to the 3′-UTR of target mRNAs to promote degradation or translational repression [88]. Moreover, considerable flexibility has been reported for mi-RNAs which are capable of targeting hundreds of genes while individual 3′-UTR mi-RNA regions may be a target for several distinct mi-RNAs [89,90]. The miRNA sequences within the 3′-UTR of human ITB genes are therefore likely to play a major role in regulating the translation of these genes within vertebrate tissues.

### 2.5. Comparative Human and Mouse ITB Tissue Expression

**Figure 5:**
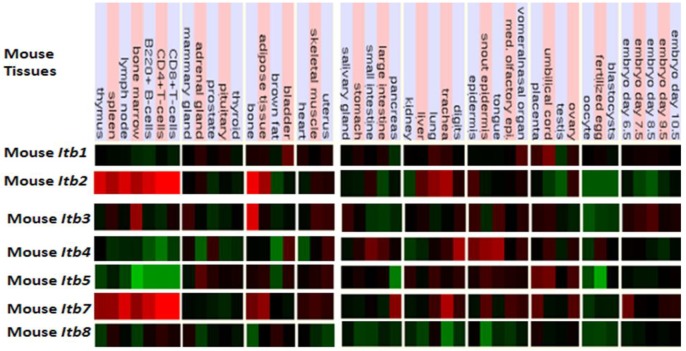
Comparative tissue expression for mouse beta integrin genes (*ITB*). Expression ‘heat maps’ (GNF Expression Atlas 2 data) (http://biogps.gnf.org) [91] were examined for comparative gene expression levels among human and mouse tissues for *ITB* genes showing high (red); intermediate (black); and low (green) expression levels; derived from mouse genome browsers (http://genome.ucsc.edu) [45].

Figure 5 presents ‘heat maps’ showing comparative *Itb* gene expression for various mouse tissues obtained from GNF Expression Atlas Data using GNF1M chips (http://genome.ucsc.edu; http://biogps.gnf.org) [91]. These data supported a broad and high level tissue expression for mouse *Itb7*, including during early embryonic development. A very high level of expression for *Itb2* and *Itb7* in bone marrow, spleen and lymphocytes are consistent with their involvement in forming the integrin receptors in blood cells [92]. The *Itb4* expression was highest in epidermal tissues and is consistent with its presence in the hemidesmosomes of these epithelial cells [93,94]. It may be noted that ITB4 pairs only with the α6 subunit forming a laminin-binding receptor providing stable adhesion of epithelial cells with the basement membrane [95,96]. Other comparisons of mouse *Itb* tissue expression indicated significant differences, including higher levels of *Itb3* expression in bone but with lower expression levels for *Itb8* in most tissues examined. Overall, mouse *Itb* tissue expressions levels were up to 3.7 times the average level of gene expression [57] which supported key roles played by these membrane receptor proteins which serve various cell adhesion roles in tissue repair, hemostasis, immune response, embryogenesis and metastasis [92]. Similar tissue distribution profiles for *ITB* gene expression were observed for human tissues, including an overall high level gene expression ranging from 0.8–6.1 times the average level of human gene expression (Table 2).

### 2.6. Evolution of Vertebrate ITB Genes and Proteins

**Figure 6:**
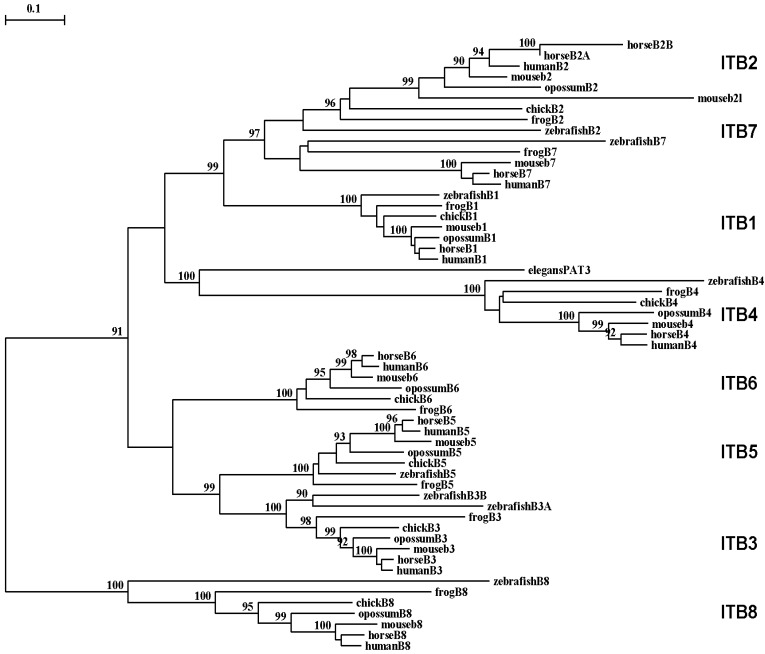
Phylogenetic tree of vertebrate beta integrin cytosolic domain amino acid sequences. The tree is labeled with the ITB name and the name of the animal and is ‘rooted’ with the *Caenorhabitis elegans* (nematode) ITB-like sequence (see Table 1). Note the 7 major clusters corresponding to the *ITB1*, *ITB2*, *ITB3*, *ITB4*, *ITB5*, *ITB6*, *ITB7* and *ITB8* gene families. A genetic distance scale is shown (% amino acid substitutions). The number of times a clade (sequences common to a node or branch) occurred in the bootstrap replicates are shown. Only replicate values of 90 or more which are highly significant are shown with 100 bootstrap replicates performed in each case.

A phylogenetic tree (Figure 6) was calculated by the progressive alignment of 61 vertebrate ITB amino acid sequences with vertebrate ITB1-8 sequences which was rooted with the *Caenorhabitis elegans* (nematode) ITB-like sequence (see Table 1). The phylogram showed clustering of the ITB sequences into groups which were consistent with their evolutionary relatedness, as well as groups for each of vertebrate ITB1–ITB8 which were distinct from the nematode ITB-like sequence. These groups were significantly different from each other (with bootstrap values of >90) and showed closer relatedness for the following *ITB* gene groupings: group 1: ITB1-ITB2-ITB7; group 2: vertebrate ITB4 with the elegans ITB-like sequence (PAT3); group 3: ITB3-ITB5-ITB6; and group 4: ITB8, which is the most distinct group in terms of its relatedness to other ITB gene families. It is apparent from this study of vertebrate ITB genes and proteins that these are ancient proteins for which a proposed common ancestor for the *ITB* genes may have predated the appearance of fish >500 millions of years ago [96].

Among the ITB integrin genes examined, the *ITB4* integrin subunit gene related most closely with the *C. elegans* (nematode) PAT3 sequence indicating that it may represent the primordial vertebrate beta integrin gene and the first to appear in the vertebrate ancestor. The ITB4 differs from other ITB subunits. It is unusually longer (1778 residues) compared with other integrin β subunits and contains a long amino-terminal (683 aa) and cytosolic (1072 aa) domains [33]. The extracellular domains of β4 subunit showed low identity (∼35%) with other β integrin subunits. Moreover, the transmembrane domain of the ITB4 subunit is poorly conserved and is exceptionally long [92,97,98].

### 2.7. Vertebrate ITB Integrins: Functional and Comparative Aspects

Different integrin beta receptor proteins are known to interact with at least 22 different ligands and matrix proteins [14,92, 99,100,101,102] that are summarized in Table 3. Of these, ITB1 pairs with the largest number of ligands (12 ligands) followed by ITB2 and ITB3 (7 ligands each), ITB5, ITB6 and ITB7 (3 ligands each), ITB8 (2 ligands) and ITB4 (1 ligand). Moreover, ITB1 pairs with the largest number of α subunits (fourteen) followed by ITB2 (four), ITB3 and ITB7 (two), and ITB4, ITB5, ITB6 and ITB8 (single α subunit). The ITB4 subunit pairs only with the ITA6 subunit forming α6β4 as the sole integrin receptor of the hemidesmosomes, a structural component, that is required for the attachment of cells with the basal lamina [103,104,105]. The genes and proteins of hemidesmosomes [107] date back to metazoans/holozoans, suggesting that the attachment of unicellular life forms on the basal lamina via the hemidesmosomes possibly initiated the formation of multicellular organisms with the evolution of other cell-cell junction components (tight, adherent, desmosmal and gap junctions). The tissue specific expression of integrin ITB subunits (Figure 6) showed that mammalian Type I hemidesmosomes are found in the epithelial cells of skin, mouth and esophagus whereas Type II hemidesmosomes are found in the intestinal epithelial cells [93]. This is consistent with the high expression of *ITB4* in epithelial cells. While the ITB4 subunit in the α6β4 integrin receptor primarily plays a role in the formation of stable adhesions of epithelial cells with laminin-332, recent studies have suggested an additional role in the migration of keratinocytes and cancer cells [106,108]. Prior to migration, keratinocytes lose their stable adhesion mediated by hemidesmosomes and migrate over collagen and then secrete a provisional matrix of laminin-332 for its motility [109]. The cancer cells also require laminin-332 to migrate [110]. It is now known that the proteolytic cleavage of laminin-332 triggers cell motility of cells via the α6β4 receptor [111]. Other evidence suggests that the migration on laminin-332 is indeed mediated by the α3β1 integrin rather than the α6β4 integrin which actually has transdominating inhibiting effects on migration mediated by the α3β1 integrin [112]. Overall these reports indicate that the ancestral role of integrin in forming stable adhesions of epithelial cells via hemidesmosome might have evolved to support migratory roles of cells by the introduction of additional integrin receptors to perform specialized functions. In this regard, the evolution of the ITB1 subunit from the primordial ITB4 may have played a significant role in influencing cell migration. The transmigration of blood cells across the endothelial layers, a highly specialized function mediated by the integrins, may be associated with the evolution of receptors αvβ3 and those formed by the association of the ITB2 subunit with αL, αM, αX or αD subunits [113,114,115,116,117,118,119].

**Figure 7:**
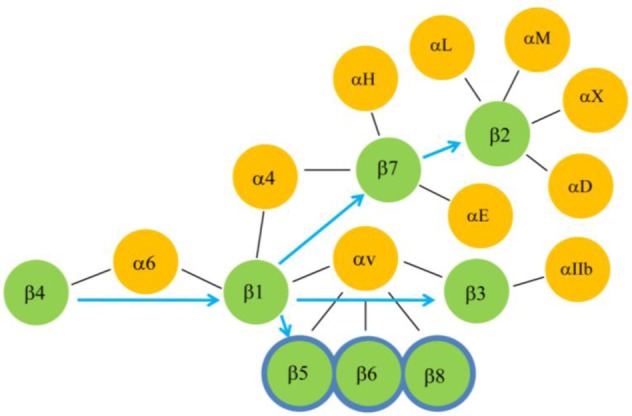
The blue arrows show the predicted evolutionary paths of β subunits from the ancestral β4 subunit. Pairings of different α and β subunits are shown by thin black lines. This concept shows two lines of evolution diverging from β1, one towards blood cell integrins consisting of β2 or β7 subunits and the other towards a cluster consisting of β5, β6, β8 and β3 subunits that are primarily expressed in tissues other than blood with exception of α IIb.β3 that is expressed also in blood platelets (see text).

The extracellular domains of both α and β subunits of a receptor interact with wide spectrum of ECM molecules (Table 3) to perform various cellular functions. This suggests that these receptors may have evolved along with the evolution of ECM molecules for performing diverse functions in the context of presence or absence of specific ligands. Consequently, the ITB1 subunit may be the most promiscuous of all of the vertebrate β subunits as it pairs with the largest number of α subunits, and these alpha/beta1 heterodimers also interact with a large number of ligands. This is consistent with the observation that ITB1 like subunits had already diverged in the earlier stages of metazoans (corals and sponges) [120]. Therefore, the clues to the evolution of different vertebrate integrin receptors may lie in their evolution to interact with different ECM molecules. However, in the absence of comprehensive information on the different domains/motifs of the ECM molecules that interact with the specific domains of different integrin receptor, further conclusions may not be derived. Nevertheless, the clues to the evolutionary proximity amongst different β subunits might be found in their ability to pair with common α subunit/s, since these β subunits are likely to preserve domain/s that determine their ability to associate with similar α subunit/s or vice versa [121]. With this notion and based on the overlapping subunit compositions of functional integrin receptors (Figure 7), it is predicted that ITB1 that shares the sole alpha subunit (α6) with ITB4, is the closest to the ancestor ITB4. The ITB1 evolved to pair with the largest number of alpha subunits (Table 3) including α4 that is shared with ITB7, and the αv subunit that is shared with ITB3, ITB5, ITB6 and ITB8. Therefore, the cluster containing ITB3, ITB5, ITB6 and ITB8, and the cluster consisting of ITB7 and ITB2 may have been derived directly from ITB1. The origin of ITB5, ITB6 and ITB8 from ITB3 (rather from the versatile ITB1) is less likely because ITB3 is the most specialized of this cluster and is expressed in both blood cells (platelets) and other cell types such as placental trophoblast and cancer cells [122,123,124]. In contrast, ITB5, ITB6 and ITB8 including ITB1 are not expressed in blood cells. The ITB2 and ITB7 subunits, that constitute solely the integrins of hematopoietic and immune system [125], and specifically ITB2 that does not share an α subunit with other ITB subunits, are likely to be the most specialized ITB subunits. The αLβ2 mediates migration of T-Cells across the endothelium (invasion or transmigration) and the α4β7expressed on memory T cells directs their trafficking to the sites of inflammation.

**Table 3:** Multiplicity and specificity of ligand binding by ITB subunits; ECM refers to extracellular matrix.

**ECM or Ligand**	**β Integrin subunit**	**Integrin Receptor/s**
Collagens	β1	α1β1, α2β1, α10β1, α11β1
E-Cadherin	β1, β7	αEβ7, α2β1
Endorepellin	β1	α2β1
Endostatin	β1	α5β1
Factor X	β2	αMβ 2
Fibronectin	β1, β2, β3, β6 and β7	α4β1, α5β1, α8β1, αvβ1, αIIbβ3, αvβ3, αvβ6, α4β7, αMβ2
Fibrinogen	β2, β3 and β5	αMβ2, αXβ2, αIIbβ3, αvβ3, αvβ5
ICAM-1, -2, -3, -5	β2	αLβ2
ICAM-3	β2	αDβ2
iC3b	β2	αMβ2, αXβ2
Laminins	β1 and β4	α1β1, α2β1, α3β1, α6β1, α6β4, α7β1,
MadCAM-1	β7	α4β7
Nephronectin (RGD)	β1	α8β1
Osteopontin	β1, β3 and β5	αvβ1, αvβ3, αvβ5
Semaphorin 7A	β1	α1β1
Tenascin-C	β1, β3 and β6	α1β1, α8β1, α9β1, αvβ3 and αvβ6
TGF-β-LAP (RGD)	β6 and β8	αvβ6, αvβ8
Tumastatin	β3	αvβ3
VCAM-1	β2 and β7	αDβ2, α4β7, αHβ7
VEGF-C, VEGF-D	β1	α9β1
Vitronectin	β1, β3, β5 and β8	αvβ1, α8β1, αvβ3, αvβ5, αvβ8, ,
Von Willebrandt Factor	β3	αIIbβ3, αvβ3,

An analysis of α subunit sharing by different ITB subunits suggests that evolution of the ITB1 subunit led to the emergence of two groups of ITB subunits, one consisting of ITB3, ITB5, ITB6 and ITB8 subunits and the other consisting of ITB7 and ITB2. This conclusion from the subunit sharing concept (Figure 7) is very similar to our phylogenetic analysis data that suggests that ITB1-ITB7-ITB2 belong to one cluster and the ITB3-ITB5-ITB6 as another cluster. A previous phylogenetic study on ITBs [16] supported ITB1-ITB7-ITB2 as one cluster and the ITB3- ITB5-ITB8 as another cluster. Therefore, two phylogenetic analyses differed by one subunit (ITB6/ITB8) in their second cluster but both found ITB4 either an outlier or an ancestral integrin subunit. The ITB8 is found to be a distinct member of ITBs in our study whereas in the previous study it was found to diverge from the ITB6 earlier in evolution. The subunit pairing concept (Figure 7), however, groups the ITB8 subunit belonging to cluster 2 of both studies together (ITB3-ITB5-ITB6-ITB8) which is consistent with a previous report [15].

## 3. Methods

### 3.1. Vertebrate ITB Gene and Protein Identification

BLAST (Basic Local Alignment Search Tool) studies were undertaken using web tools from the National Center for Biotechnology Information (NCBI) (http://blast.ncbi.nlm.nih.gov/Blast.cgi) [127]. Protein BLAST analyses used human and mouse ITB amino acid sequences previously described (Table 1). Non-redundant protein sequence databases for several vertebrate genomes were examined using the blastp algorithm, including human (*Homo sapiens*) [128]; horse (*Equus caballus*) [129]; mouse (*Mus musculus*) [130]; opossum (*Monodelphis domestica*) [131]; chicken (*Gallus gallus*) [132]; frog (*Xenopus tropicalis*) (http://genome.jgi-psf.org/Xentr3/Xentr3.home.html); zebrafish (*Danio rerio*) (http://www.sanger.ac.uk/Projects/D_rerio/); and nematode (*Caenorhabditis elegans*) (http://genome.ucsc.edu/). This procedure produced multiple BLAST ‘hits’ for each of the protein databases which were individually examined and retained in FASTA format, and a record kept of the sequences for predicted mRNAs and encoded ITB-like proteins. These records were derived from annotated genomic sequences using the gene prediction method: GNOMON and predicted sequences with high similarity scores for human ITB. Predicted ITB-like protein sequences were obtained in each case and subjected to analyses of predicted protein and gene structures.

BLAT analyses were subsequently undertaken for each of the predicted ITB amino acid sequences using the UCSC Genome Browser (http://genome.ucsc.edu/cgi-bin/hgBlat) [45] with the default settings to obtain the predicted locations for each of the mammalian *ITB* genes, including predicted exon boundary locations and gene sizes. Structures for human and mouse isoforms (splicing variants) were obtained using the AceView website to examine predicted gene and protein structures (http://www.ncbi.nlm.nih.gov/IEB/Research/Acembly/index.html?human) [57].

### 3.2. Prediction of Signal Peptide Sequence and the Secondary Structure of Human Vertebrate ITB Proteins

FASTA sequence of different human β integrin amino acid sequences were subjected to SignalP 3.0 Server (http://www.cbs.dtu.dk/services/SignalP) [133] to determine the number of amino-acids and the predicted secondary structures in the *N*-terminal end of the ITGB isoform involved in the formation of the signal peptide. The secondary structures of each signal peptide were determined using a SWISS-MODEL workspace (http://swissmodel.expasy.org) [134].

### 3.3. Predicted Structures and Properties of the Cytoplasmic Domains of Vertebrate Beta Integrins

Predicted secondary and tertiary structures for the predicted cytoplasmic domains of vertebrate ITB-like proteins were obtained using the SWISS MODEL web tools [134]. The tertiary structures of the cytoplasmic tails for human ITB1 [residues 1–36], ITB2 [residues 1–47] and ITB7 [residues 1–38] were predicted using a model (PDB: 3g9wC) for human ITB1 [135]; while the reported structure for human ITB3 [136] (PDB:1m8oB) served as the reference for human ITB3 (residues 1–47), ITB5 (residues 1–40) and ITB6 (residues 1–45) tertiary structures, and the human ITB4 structure (PDB: 2yrzA) [137] for human ITB4 (residues 906–1007). Theoretical isoelectric points and molecular weights for vertebrate ITB-like proteins were obtained using Expasy web tools (http://au.expasy.org/tools/pi_tool.html).

### 3.4. Comparative Human Beta Integrin (ITB) Expression

The UCSC Genome Browser (http://genome.ucsc.edu) [45] was used to examine GNF Expression Atlas 2 data using various expression chips for human ITB genes (http://biogps.gnf.org) [91]. Gene array expression ‘heat maps’ were examined for comparative gene expression levels among human and mouse tissues showing high (red); intermediate (black); and low (green) expression levels.

### 3.5. Comparative CpG Islands, Transcription Factor Binding Sites (TFBS) and microRNA Sequences of Human Beta Integrin Genes (ITB)

The UCSC Human Genome Browser (http://genome.ucsc.edu) [45] was used to examine the comparative location, number and sequences for human CpG islands, transcription factor binding sites (TFBS) and microRNA sites located in the 3′-untranslated region (UTR) of human *ITB* genes in association with the TargetScan website (http://www.targetscan.org).

### 3.6. Phylogeny Studies and Sequence Divergence

Alignments of vertebrate ITB-like and nematode (*Caenorhabditis elegans)* PAT3 protein sequences were assembled using BioEdit v.5.0.1 and the default settings [137]. Alignment ambiguous regions were excluded prior to phylogenetic analysis yielding alignments of 370 residues for comparisons of vertebrate ITB sequences with the nematode PAT3 (beta-integrin homolog) sequence (Table 1). Evolutionary distances were calculated using the Kimura option [138] in TREECON [139]. Phylogenetic trees were constructed from evolutionary distances using the neighbor-joining method [140] and rooted with the nematode PAT3 sequence. Tree topology was reexamined by the boot-strap method (100 bootstraps were applied) of resampling and only values that were highly significant (≥90) are shown [141].

## 4. Conclusions

Bioinformatic analyses of the integrin genes and proteins in vertebrates revealed a high degree of diversity in terms of their chromosome locations, alternate splicing, transcriptional and post-transcriptional regulations, and tissue specific expressions. Results suggested that the evolution of integrins within vertebrates followed a divergent path for these genes and protein structures but with common functions specializing towards adhesion, migration and transmigration of cells in succession. Our phylogenetic analysis revealed for the first time that *ITB4* (encoding the β4 integrin) is the most likely ancestral form of integrin β-like genes. This subunit has inherited the ancestral role for β-integrins in forming simple adhesions (hemidesmosomes) in vertebrate cells similar to unicellular organism and is also involved in the migration of transformed (cancer) cells [7]. The subunit sharing analysis of ITB subunits reveals that β2 and β7 subunits that are expressed only in the cells of hematopoietic and immune system are possibly the most specialized forms of integrins.
